# The roles, responsibilities and practices of healthcare assistants in out-of-hours community palliative care: A systematic scoping review

**DOI:** 10.1177/0269216320929559

**Published:** 2020-06-15

**Authors:** Anne Fee, Deborah Muldrew, Paul Slater, Sheila Payne, Sonja McIlfatrick, Tracey McConnell, Dori-Anne Finlay, Felicity Hasson

**Affiliations:** 1 Institute of Nursing and Health Research, Ulster University, Newtownabbey, UK; 2 International Observatory on End of Life Care, Lancaster University, Lancaster, UK; 3 Marie Curie Hospice, Belfast, Belfast, UK; 4 School of Nursing and Midwifery, Queen’s University Belfast, Belfast, UK

**Keywords:** Healthcare assistant, after-hours, palliative care, community, adult, home care, scoping review

## Abstract

**Background:**

Access to community palliative care ‘out-of-hours’ – defined as care provided after the normal hours of work – is advocated globally. Healthcare assistants, who provide care under the direction of a qualified professional, are increasingly employed to help deliver such care, yet there is a little understanding regarding their role, responsibilities or contribution.

**Aim:**

The aim of this study was to identify the roles, responsibilities and contributions of healthcare assistants in out-of-hours community palliative care.

**Design:**

Scoping review

**Data sources:**

Five bibliographic databases (CINAHL, MEDLINE, EMBASE, PsycINFO and Scopus) and grey literature were searched using a predefined search strategy. The review was conducted in accordance with the Preferred Reporting Items for Systematic Reviews and Meta-Analyses extension for Scoping Reviews statement.

**Results:**

The search yielded six papers using quantitative, qualitative and mixed methods. Results highlighted a lack of recognition of the role and contribution of healthcare assistants. A concurrent theme was that healthcare assistants continually monitored and responded to patient’s and family’s physical and emotional needs; there was also self-reported evidence indicating patient and family benefit, such as maintaining a sense of normality and support to remain at home.

**Discussion:**

This review highlighted a dearth of evidence relating to the healthcare assistant role in out-of-hours palliative care. Limited evidence suggests they play a role, but that it is hidden and undervalued. Such invisibility will have a significant impact on the planning and delivery of out-of-hours palliative care. Future research is needed on role development for the benefit of patients and caregivers.

**What is already known about the topic?**

- Various definitions of out-of-hours exist internationally.
- Healthcare assistants are an important component of the healthcare workforce across all clinical settings and specialisms, including palliative care.
- Out-of-hours community palliative care and the role of the healthcare assistant in the delivery of this service are neglected areas of research.

**What this paper adds?**

- The review highlights a lack of previous research in relation to the role and contribution of healthcare assistants to community-based out-of-hours palliative care.
- Some evidence in the review suggests that healthcare assistants play an integral role in the delivery of end of life and community palliative care to patients and families.
- The role of healthcare assistants is currently hidden and undervalued.

**Implications for practice, theory or policy**

- A need exists for further research into why the role of healthcare assistant is undervalued despite evidence about the importance of contributions made by healthcare assistants in community palliative care.
- A lack of policy relating to the healthcare assistant role as evidenced in this review may imply a lack of acknowledgement of this role by policymakers, potentially impacting on the recognition of the healthcare assistant role within an out-of-hours context.

## Introduction

Globally, evolving demographics and changing epidemiology of diseases have increased the demand for palliative care, with an estimated 40 million people living in need.^1^ Most people spend their last year of life at home, with many wishing to die there.^2–4^ Consequently, the necessity to improve access to community/home-based care systems has been advocated.^1,5^ However, supporting patients to fulfil their wishes and prevent admission to hospital is a significant task, especially considering that almost two-thirds of the week is spent ‘out-of-hours’ when unexpected deterioration may occur.^6,7^ Variations in the definition of out-of-hours exist internationally^8^ For example, in the United Kingdom, on weekdays, the out-of-hours period is between 18.30–08.00 h, while in Belgium, Switzerland and Germany, on weekdays, the out-of-hours period begins at 19.00^8^ and in Italy and France, it begins at 20.00.^8^

Research in the United Kingdom suggests that 30% of patients have contact with out-of-hours community palliative care services in the last days of their life, with such care recognised as integral to integrated palliative care^9–11^ Yet the delivery, organisation and effectiveness of such services are often overlooked by research and policy directives.^8,12^ These factors may be attributed to the significant variations in out-of-hours palliative care provision models which exist internationally and nationally.^8,13^ Evidence suggests that models of out-of-hours care vary in title, service design and service providers; spanning primary, secondary and voluntary sectors.^8^ Such services are delivered by members of multidisciplinary teams. Even though variations in composition of these teams across many European countries have been noted,^14^ the teams predominately comprise specialist and generalist doctors and nurses, allied health professionals and healthcare assistants, all of whom play a key role in delivery of the service. However, health workforce shortages and financial constraints have led to an increasing reliance on healthcare assistants to deliver care.^15,16^ Healthcare assistants have been found to be a vital component of the healthcare workforce across all clinical settings and specialisms, including palliative care, yet their role and value remains hidden.^17^ Internationally, the title used to refer to the healthcare assistant role varies from nurse care workers in China^18^ to personal care assistants and nursing assistants in Australia.^19^ Across Europe and within individual countries, a range of titles are used; however, the most common title is healthcare assistant.^20^ Healthcare assistants commonly work under the supervision of registered professionals, however, are not regulated, nor subject to any formal national training standards. They have been found to play an integral role in providing community palliative care within routine working hours,^21–23^ often working in isolation, at a distance from qualified supervision.^24^

Evidence suggests that healthcare assistants are key in the care provision of chronic and end-of-life care,^25^ providing clerical support,^16^ personal care and direct clinical care.^17,26–29^ In palliative care, delivered during normal working hours, they are mainly associated with assisting with activities of daily living. However, their role has evolved to include wound care, pressure care, mobilisation and ambulation, assistance with medication, bowel and ostomy care and vital signs/blood sugar monitoring assistance.^30,31^ Often, they are the first to recognise and alert professionals to patient changes^32,33^ and are relied upon for their observations and reports for registered professionals to develop and implement a plan of care for the patient and their family.^34^ In the home, within normal working hours and in the out-of-hours period, healthcare assistants have been found to act as go-between with the family caregiver and the healthcare professional, provide support and guidance to caregivers and patients on what to expect during an illness and provide a reassuring presence up to and at the point of death.^24,26^ Despite this, healthcare assistants are rarely recognised as an integral team member.^35–37^ Moreover, concerns have been raised that limited training^38^ has potentially resulted in a lack of knowledge and preparation to care for patients at the end of life or respond to death and dying.^29,39^

The role of registered professionals in out-of-hours services, such as general practitioners (GPs), district nurses and specialist palliative nurses, has been reported.^39–43^ Yet the current evidence base suggests a paucity of research focusing on the healthcare assistant in out-of-hours community palliative care.^24^ Their role, contribution and impact in the out-of-hours period remain largely unknown. The aim of the current review was, therefore, to identify the roles, responsibilities, contributions and policies relating to healthcare assistants in out-of-hours community palliative care.

For the purpose of this review, ‘out-of-hours community palliative care’ refers to care provided for adults in the patient’s home by *any* healthcare team (including, but not limited to, hospice, nursing homes, residential homes and community care teams).

## Method

In this review, established scoping review methodologies were used.^44,45^ This approach enabled the inclusion of diverse methodologies and is considered suitable for use in new and emerging areas.^44,46^ A scoping review is ‘a form of knowledge synthesis, which incorporates a range of study designs to comprehensively summarise and synthesise evidence with the aim of informing practice, programmes and policy and providing direction to future research priorities’.^47^ In compliance with the scoping review guidelines, a review protocol was not published or the study registered with PROSPERO.^45,47^ Furthermore, unlike systematic reviews, quality appraisal of the evidence was not performed aligning with the premise that scoping reviews do not attempt to assess the ‘weight of evidence’.^44,45,48^ Specifically, the stages of this scoping review were as follows: (1) identifying the research question, (2) identifying relevant studies, (3) study selection, (4) charting the data and (5) collating, summarising and reporting results. The review was conducted in accordance with the Preferred Reporting Items for Systematic Reviews and Meta-Analyses (PRISMA) reporting guidelines for scoping reviews (PRISMA-ScR)^49^ which enabled standardised reporting and aids in the replicability of the review.

### Stage 1: identifying the research question

Research questions were developed based on discussions within the research team and key stakeholders with specialist healthcare background and/or expertise in the topic area.

1. What are the roles, responsibilities and contribution of healthcare assistants to out-of-hours community palliative care?
2. What does existing policy tell us about the role and/or practice of healthcare assistants locally, nationally and internationally in out-of-hours community palliative care?

For the purpose of this review, the common definitions of palliative care and the UK standard definition of out-of-hours care guided this search (see Box 1).

**Box 1.** Definitions.

**Palliative care** – ‘an approach that improves the quality of life of patients and their families facing the problem associated with life-threatening illness, through the prevention and relief of suffering by means of early identification and impeccable assessment and treatment of pain and other problems, physical, psychosocial and spiritual’.^1^ **Out-of-hours care** – ‘The out-of-hours period is from 6.30 pm to 8 am on weekdays and all day at weekends and on bank holidays’^2^ (http://www.gpoutofhours.hscni.net). **Healthcare assistant** – ‘Healthcare assistants provide routine personal care, support and assistance with activities of daily living to patients and residents in a variety of healthcare settings. They assist patients with personal, physical mobility and therapeutic care needs as per established care plans and practices, and generally under the direct supervision of medical, nursing or other health professionals or associate professionals.’^50^

### Stage 2: search methods

Five online databases (MEDLINE, CINAHL, EMBASE, PsycINFO and Scopus) were systematically searched from January 2008 to November 2018 and updated in December 2019, in liaison with a subject librarian.

A previous systematic review on the role of healthcare support workers in providing palliative and end-of-life care in the community considered the evidence between 1990 and May 2011.^17^ One of the key challenges was the vast array of phrases used to describe this role.^17,35^ Given the limited number of research studies in this area, combined with the complexity of identifying all key terms for the healthcare assistant role, a broader approach to the database searches was utilised.

The search strategy was informed by a recognised palliative care filter to improve accuracy.^51^ The search combined key terms and concepts using Boolean logic and operators. To expand the search, truncation was employed to capture variations of words (e.g. plural/singular and American/British spelling). For palliative care, a search strategy was initially developed for MEDLINE using terms, such as exp Terminal Care/ OR hospice*.mp. OR end of life.af. OR terminally ill.mp. OR palliat*tw. OR Palliative Care/ OR exp palliative therapy (see Table 1). This was then modified for the other databases.

**Table 1.** Search strategy, MEDLINE.

1. care of dying.mp. 2. care of the dying.mp. 3. dying.mp. 4. edge of life.mp. 5. end of life.mp. 6. end-of-life care.mp. 7. hospice*.mp. 8. hospice care.mp. 9. palliative care.mp. 10. palliat*.mp. 11. palliative.mp. 12. palliative care services.mp. 13. palliative therapy.mp. 14. supportive care services.mp. 15. support care.mp. 16. supportive care.mp. 17. terminal*.mp. 18. terminal care.mp. 19. end of life care.mp. 20. 1 or 2 or 3 or 4 or 5 or 6 or 7 or 8 or 9 or 10 or 11 or 12 or 13 or 14 or 15 or 16 or 17 or 18 or 19 21. after hours.mp. 22. after-hours.mp. 23. out of hours.mp. 24. out-of-hours.mp. 25. out of hours services.mp. 26. weekend services.mp. 27. 24-7.mp. 28. 21 or 22 or 23 or 24 or 25 or 26 or 27 29. 20 and 28 30. limit 29 to year:"2008-Current" 31. limit 30 to English language

Hand searches were also performed from the reference list of relevant studies; key journals (e.g. British Journal of Healthcare Assistants) and key authors were contacted for further relevant studies. Articles published before 2008, not published in English, reporting on care provided outside the patient’s home, focussing on clinical outcomes only or reporting on the perspective or experience of the family and caregiver, were excluded.

Grey literature searches were undertaken of PhD theses and dissertations (British library Ethos, ProQuest Dissertations and Theses Global, Theses Canada and DART – Europe E-Theses Portal), clinical trials register (US Clinical Trials), grey literature databases (OpenGrey and OpenDOAR), systematic reviews register (PROSPERO) and networks (The Knowledge Network – formerly NHS e-library). In addition, websites of relevant organisations (e.g. The King’s Fund, Macmillan, UK Royal College of Nursing, Marie Curie and European Association of Palliative Care) were also searched to identify empirical evidence on the roles, responsibilities and practices of healthcare assistants. The same search terms were applied to grey literature searches. A separate search strategy was employed to identify literature pertaining to policies that describe the roles, responsibilities or practices of healthcare assistants within out-of-hours community palliative care. The following search terms were entered into Google and websites of relevant organisations (e.g. Royal College of Nursing): ‘healthcare assistant’, ‘palliative care’, ‘community setting’ and their various synonyms in combination with ‘policy’, ‘guideline’, ‘action plan’ and ‘strategy’. University online library catalogues for policy were searched using the same strategy.

### Stage 3: inclusion and exclusion criteria

It has been advocated that the research question and purpose should guide the decision-making around the scope of the study.^44^ Articles were selected for review and inclusion if they met pre-determined eligibility criteria (see Table 2).

**Table 2.** Eligibility criteria.

Inclusion criteria
Report the role, responsibility or contribution of the healthcare assistant or equivalent role. Report on the model of out-of-hours palliative care provision, including the system, the process or the outcome. Report out-of-hours care provided for adults, in the patient’s home by any healthcare team (including, but not limited to, hospice, nursing homes, residential homes and community care teams). Are published in the English Language. Have available full text (the authors of the study will be emailed a maximum of twice to ask for full text versions if it is not freely available online. If no response is received, the article will be excluded). Published between January 2008 and December 2019. Report empirical research of any methodology or design (e.g. randomised controlled trial (RCT), qualitative research).

Eligibility criteria were independently applied to titles and abstracts of articles retrieved from the database searches by two researchers (D.M. and F.H.). If relevance to the research question was unclear at the abstract level, the full text was reviewed. Following the execution of the search strategy, all citations were exported to Mendeley Desktop Reference Manager for examination and selection. Any disagreements that arose between the reviewers were resolved through discussion or through the involvement of a third reviewer (A.F.). Articles that were considered eligible after title/abstract screening were obtained and screened as full texts. At this stage, the following additional exclusions were applied to full texts: (1) not inclusive of the healthcare assistant role, (2) findings focussed on patient/caregiver data and (3) not relating to out-of-hours care. If studies did not specify all the inclusion criteria (e.g. 24/7), but referred to this in their findings, then the author was contacted for clarification. Grey literature was independently screened by two researchers (F.H. and A.F.). Academics with expertise in palliative care and the healthcare assistant workforce reviewed final selected articles to ensure relevance to the research question.

### Stage 4: charting the data

A data charting form was developed by identifying variables that corresponded with the research questions about roles, responsibilities, contribution and policy, along with information, including author(s), year of publication, study location, aims and objectives, methods and main findings (see Table 3). Data were extrapolated using an adapted data extraction form.^57^ Two authors (D.M. and F.H.) used the data extraction form, independently extracted data from all studies and met to determine whether their approach to data extraction was consistent with the research question and purpose.^41^

**Table 3.** Data extraction table.

Author, year and location	Aims and objectives	Sample	Design and methods	Findings
Carlebach,^52^ 2010 UK	To report an evaluation of an out-of-hours service operating in one primary care trust in North East England, focusing on the use of a telephone support service backed by domiciliary visits by specialist Palliative Care nurses. Telephone service is staffed by qualified nurses (2x full-time, 2x part-time) and healthcare assistants (2x full-time, 2x part-time).	Audit of calls received by the telephone service and 27 interviews with patients, carers and Health Care Professionals (District Nurses, Macmillan team and General Practitioners).	Service evaluation. Activity data and qualitative data.	Results revealed that staff, patients and carers appreciated being able to telephone the service (reactive). In addition, carers felt particularly well supported by the service staff who proactively telephoned them on an agreed basis as part of the highly individualised telephone monitoring scheme. Such services support the call for the creation of a whole system approach for both palliative care patients and their carers.
Butler,^53^ 2012 UK	To contribute to the development of the evidence base on the consequences and costs of hospice rapid response teams, compared to usual care. Outcome measure: dying in preferred place of death.	NA	(Protocol) Pragmatic quasi-experimental controlled trial. Evaluation (preferred place of death, carer QoL, interviews and service utilisation cost).	NA
Gage,^54^ 2015 UK	(1) To compare the characteristics of rapid response service users and non-users, (2) explore differences in the proportions of users and non-users dying in the place of their choice and (3) monitor the whole system service utilisation of users and non-users and compare costs.	All hospice patients who died in their preferred place of death during an 18-month period (1527 eligible).	Quantitative. Randomised stepped wedge design.	Delivery of a Rapid Response Service – trained by hospice and supported by full multidisciplinary Palliative Care team. Contributed to a ‘good death’. Chances of dying in the preferred place were enhanced 2.1 times by being an Rapid Response Service user, compared to a non-user, and 1.5 times by having a co-resident carer, compared to living at home alone or in a care home. Total service costs did not differ between users and non-users, except when referred to hospice very close to death (users had higher costs).
Seow,^55^ 2017 Canada	To explore similarities in care practices among effective and diverse specialist teams to inform the development of other community-based teams.	In total, 78 providers and administrators from 11 distinct community-based specialist palliative care teams from Ontario.	Qualitative semi-structured interviews, inductive analysis.	Key themes: First, the distinct models of care were generally summarised into three models: primary care and specialist providers either collaborated by transferring, sharing or consulting in care. Second, teams explicitly or implicitly followed seven common care practices related to specialised expertise 24/7, intrateam communication, timeliness, physical symptom and psychosocial–spiritual management, education, peace and fulfilment and advocacy for patient preferences. Third, all teams emphasised the importance of team building, even more than using clinical tools and processes. No specific reference to role or responsibility of healthcare assistants.
Seow,^56^ 2018 Canada	To examine how a variety of home-based specialised palliative care teams created and grew their team over time and to identify critical steps in their evolution.	In total, 15 specialised palliative care teams from Ontario, including nurses, physicians, personal support workers, spiritual counsellors and administrators.	Semi-structured interviews, grounded theory approach.	Four stages in team evolution: Inception, start-up (*n* = 4 teams), growth (*n* = 5) and mature (*n* = 6). In the inception stage, a champion provider was required to leverage existing resources to form the team. Start-up teams were testing and adjusting care processes to solidify their presence in the community. Growth teams had core expertise, relationships with fellow providers and 24/7 support. Mature teams were fully integrated in the community, but still engaged in continuous quality improvement. No specific reference to role of healthcare assistants.
McPherson,^24^ 2019 Canada	To identify the types and frequencies of tasks performed by unregulated care providers (UCPs) in home-based palliative care to older clients (> 65 years) and their families and to describe UCPs’ engagement in care, and barriers and facilitators to their work.	Chart review of UCP tasks (*n* = 66); Progress notes from client charts (*n* = 85); UCP interviews (*n* = 10).	A mixed method approach was used comprising a quantitative retrospective chart review of UCPs’ tasks; qualitative content analysis of progress notes from clients’ charts and thematic analyses of in-depth interviews with UCPs.	The findings indicated that although a significant proportion (63%) of the 13,558 UCP tasks identified were directed towards meeting clients’ physical care needs, their presence in the home, made UCPs an important source of information on the client’s condition; observing and appraising the situation. Furthermore, the nature of their work and frequent interactions with clients and families also presented opportunities for UCPs to provide emotional support; a role UCPs felt was integral to their work.

### Stage 5: collating, summarising, and reporting the results

To ensure rigour, data were imported into QSR Nvivo 12 for management and refinement.^58^ An inductive approach to analysis was adopted to enable a broad exploration of the healthcare assistant role to be identified without being prescribed by any pre-existing theoretical constraints. Each paper was analysed using thematic analysis, where papers were read, coded and themed.^59^ Studies were systematically compared for common and recurring findings to establish similarities and differences, and data were organised thematically into key categories.^44,45,60^ Codes and themes based on key aspects of the research question were initially identified by one researcher (A.F.) and confirmed by a second author (F.H.). To ensure credibility, resulting codes and themes were discussed and checked for reliability through continuous peer review within the research team.^58^

## Findings

### Search outcome

**Figure 1.**
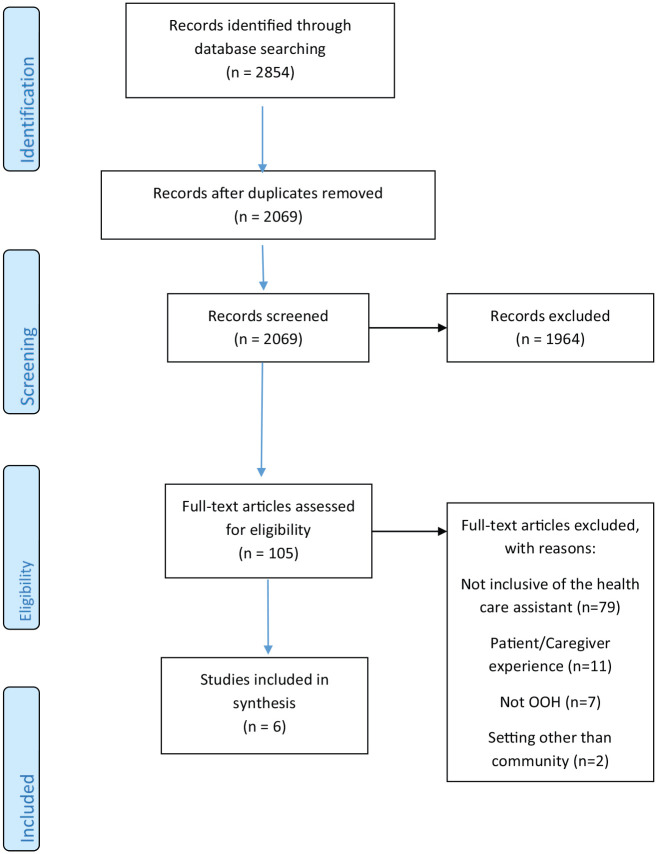
PRISMA.

The initial search retrieved 2854 articles across all databases. Removal of duplicates resulted in 2069 unique articles. After title and abstract screening, 1964 were excluded, resulting in 105 articles which were subject to full text review. Reasons for exclusion at this stage included; not inclusive of the healthcare assistant role (*n* = 79), not focused on out-of-hours care (*n* = 7), contained patient and carer data only (*n* = 11) and included settings other than community (*n* = 2) (see Figure 1). Six studies were included in the final review. The search of additional reference lists or grey literature did not result in any additional studies. Of note, no specific policy relating to the role or practice of the healthcare assistant in out-of-hours care in the community representing international, national or regional sources was identified from the search. Policies that do exist refer to the healthcare assistant role in palliative care generically and therefore outside the criteria for inclusion.

### Study characteristics

From the six papers, three were from the United Kingdom^54,53,52^ and three from Canada.^24,56,55^ Three studies used qualitative methods,^52,56,55^ one used mixed methods^24^ and two used quantitative methods.^53,54^ Study samples comprised community-based specialist palliative care teams (including healthcare assistants),^52,56^ rapid response community service (delivered by healthcare assistants with hospice multidisciplinary support),^53,54^ telephone support services (staffed by healthcare assistants and nurses)^52^ and healthcare assistants only (referred to as unregulated care providers).^24^

There was only one study that specifically focused on the impact of the healthcare assistant role within out-of-hours services.^24^ Others reported on the generic role of healthcare assistants within palliative multidisciplinary care teams, or on the out-of-hours model, with the team as a secondary focus. The papers in this review used qualitative, quantitative and mixed methods approaches; there were no randomised controlled trials, longitudinal or experimental studies to evaluate the efficacy of out-of-hours services or contribution of healthcare assistants.

### Summary of themes

Thematic data analysis^59^ resulted in the identification of two overarching themes: (1) roles and responsibilities and (2) contribution to care.

#### Theme 1: roles and responsibilities

Two papers focussed primarily on the role of healthcare assistants.^24,53^ Although one study reported on the role of a team comprising only healthcare assistants, it was not clear whether the ‘hands-on’ care provided was by this team or by other community-based services (i.e. GPs, district nursing services).^53^ Another study in Canada described the specific role of Unregulated Care Providers (UCPs – equivalent of HCAs) within a palliative care team.^24^ The lead author of this study confirmed that this research also included the out-of-hours period. In this study, authors used a mixed methods approach to review charts of healthcare assistant tasks (*n* = 66), notes from patient charts (*n* = 85) and undertook interviews with healthcare assistants (*n* = 10), resulting in the categorisation of key responsibilities from the patient, caregiver and team perspective.^24^ Patient-centred tasks included activities of daily living, such as bathing and personal care, transfers/ambulation and dressings. Authors also reported on the role and support provided to the caregiver, such as ‘helping out’ (including cleaning and meal preparation), signposting to other support services, and providing social support to the patient, caregiver and wider family circle. It was recognised that a key role of the healthcare assistant was one of being present with the patient and caregiver. This encompassed not only being physically present within the home, or offering emotional support, but also building relationships. Authors emphasised these relational aspects of the healthcare assistant’s role were crucial for meeting patients and family’s emotional needs.^24^ The frequency of healthcare assistant’s visits resulted in familiarity with patients and families and facilitated monitoring of patients and family status. Consequently, healthcare assistants played a role in ongoing monitoring and reporting back to the multidisciplinary teams.^24^

In four other studies, even though healthcare assistants were included as part of the team, their role was not distinguishable from the roles of other team members, and study findings related to the role and responsibility of the team collectively.^52,54,55,56^ Of note, was responding to pain and symptom management and responding rapidly to crises in patients’ homes.^55^ However, the healthcare assistant’s specific role in symptom management or pain control was not reported. For example, in the United Kingdom, one study described how the rapid response service, delivered solely by experienced healthcare assistants, trained by the hospice and supported by the hospice multidisciplinary team, was responsible for responding to 24/7 crisis in patients’ homes.^54^ While the specific responsibilities of the healthcare assistant role within this service were not the focus of the paper, it did state that ‘Patients’ needs and prognosis, and family circumstances are assessed, including patient/family preferences. Hands-on care is provided in coordination with other community services’.^54^

Within this theme, all articles suggested that teamwork and mutual support were important for service delivery, thus all team members had a responsibility for this.^24^ In their exploration of common care practices among community-based palliative care teams, one study described the impact of this support as ‘Belonging to a team also provided members with moral support. This would help individuals to avoid burnout and compassion fatigue, as well as, enable the team to sustain and grow their model’.^56^ Another study underpinned the significance of moral support between team members to describe how healthcare assistants experienced the importance of teamwork for the clients benefit.^20^ A healthcare assistant in the study commented: ‘Everything intertwines. You are a team; you can’t do it by yourself. If you have a client, that person for instance will have bed sores and the sore needs to be dressed. You have to communicate and let the nurse know that this person needs attention in this area. So, you are in every way 100% part of the team’.^25^

#### Theme 2: contribution to care

The contribution of healthcare assistants to the care provided was reported by two studies.^24,53^ This theme described how healthcare assistants contributed to care within the palliative care service, either as part of a palliative care team or individually. Data within the theme suggested that their contribution to care could potentially make a direct or an indirect difference. In one of the selected studies, authors examined the impact of a rapid response service on preferred place of death.^53^ Reviews of charts revealed that accessing the service doubled a patient’s chance of dying in their preferred place. By contrast, another study noted contributions to care that, although making a difference, were more of an indirect contribution. Providing care of an indirect nature, such as housework and cleaning, enabled normality to be maintained in the home and the family to concentrate on being with the patient. ‘Participants recognized that through the provision of care, they could make a difference by treating clients with respect, honouring clients’ and families’ preferences, and providing physical comfort through interventions’.^25^

## Discussion

To the best of our knowledge, this is the first scoping review to systematically examine the role, responsibilities and contribution of healthcare assistants in out-of-hours palliative care and in policy. This is reflected in the small number of included studies and is consistent with reports of a dearth of evidence regarding out-of-hours services,^8^ the healthcare assistant role in community palliative care^26,28^ and the healthcare assistant role in out-of-hours palliative care services in the community.^61^ While reports of the role and contribution of healthcare assistants are steadily growing across clinical settings and community-based palliative care services within day-time hours, in the current review, the inclusion of ‘out-of-hours’ significantly limited search results. For example, in MEDLINE, variations of ‘palliative’ and ‘end-of-life’ care services resulted in 627,542 hits. Variations in ‘out-of-hours’ terms resulted in 6119 hits. A combination of these terms resulted in 282 hits.

While limited, findings in the current review suggested that healthcare assistants played an integral role in out-of-hours community palliative care. However, the contribution varied from a central role to part of a multidisciplinary team, depending on the service model, and making comparisons between the evidence was difficult due to cultural and health system differences. Regarding the healthcare assistant role, a concurrent theme in the literature was that healthcare assistants delivered, monitored and responded to patient and family physical and emotional needs.^24^ Given that the relational aspects of the healthcare assistant role^24^ are often unrecognised and undervalued,^17^ it is important to highlight the difference made by healthcare assistants. For example, some evidence in the current review suggested that the role of healthcare assistants may have made a difference to patients and families in terms of supporting patients to remain at home and maintain a sense of normality.^24^ This was consistent with findings of previous research outside of the out-of-hours period.^17,26,53^

The current review also alluded to the healthcare assistant’s involvement in symptom management.^56^ However, specific details relating to their scope of practice, levels of education, supervision and professional standards underpinning this responsibility were lacking. Although not specifying out-of-hours, findings from a qualitative study in the United Kingdom (*n* = 14) indicated that due to comprehensive knowledge of nursing home resident ‘norms’, healthcare assistants were often the first to notice symptoms of pain in dementia patients receiving palliative care.^32^ In addition, in the current review, it was emphasised that often healthcare assistants undertook additional duties that were over and above their formal role, such as housework or laundry.^24^ Although this was suggested by previous authors,^29^ it was not clear why this occurred. A possible explanation may be that helping with household tasks may have helped to build family relationships^26^ but could also indicate an absence of family carers or paid social care support. However, this may also point to inconsistencies around roles and responsibilities, and unclear expectations about the healthcare assistant role from other team members or family members.

The role of the healthcare assistant role is further complicated when considering the funding models operated for personal care. For example, within England, the contribution of domiciliary homecare workers (also termed social carers) who are employed by other independent social care agencies, (i.e. not under the supervision of the palliative care team) has been recognised as an additional factor in support provided within this home.^61^ These workers may be fulfilling similar roles to some healthcare assistants, for example, by aiding with activities of daily living, such as personal care and assisting with meals. However, it has been suggested that a lack of palliative care training could mean that these homecare workers may not be as sensitive to the family’s needs as their counterparts (who are part of a palliative care team),^62^ thus potentially adding complexity to care provided.

Studies examining the healthcare assistant role within *day-time* community-based palliative care have also identified challenges and support needs of healthcare assistants in this context. Among these were training deficits, and the need for additional support and supervision,^29^ emotional attachment, role ambiguity and inadequate training,^17^ and balancing the emotional element of the work with practical responsibilities.^26^ The extent to which these issues could also be applied to the out-of-hours period, and whether the out-of-hours context brings additional needs of healthcare assistants needs to be examined in more depth. In the current review, findings revealed that challenges involved for healthcare assistants included incompatibility between healthcare assistants’ competencies and patients’ needs; emotional attachment, undertaking tasks beyond their remit, working in isolation without colleagues for support and the provision of emotional support to families dealing with loss (which was not recognised by any formal process).^24^

## What this review adds to existing knowledge

This review improves understanding about the role and contribution of the healthcare assistant in the delivery of out of-hours palliative care in the community. Review findings have implications for providers of palliative care, specifically within an out-of-hours context. In particular, the dearth of evidence about the role and contribution of healthcare assistants within community-based palliative care has raised important questions. For example, there is an increased international emphasis on care in the community, and out-of-hours care has been reported as contributing to good integrated palliative care and integral to facilitating death at home.^8,56^ Given that healthcare assistants play an important role in providing community-based palliative care,^21,24^ questions remain as to why they are rarely recognised as integral team members^35–37^ and mainly unrecognised in policy. In comparison with the roles of other multidisciplinary palliative care team members reported in the literature,^40,41,43^ evidence from the current review suggests that the role and contribution of healthcare assistants remains largely invisible. Findings of this review revealed a significant gap in the literature and point to a need to further examine reasons why the role of healthcare assistants in an out-of-hours context was not valued and why policy failed to recognise their contribution.

## Strengths and limitations

This systematic scoping review has several strengths in terms of study design. First, to ensure relevant studies were identified, an extensive search was performed across multiple databases and the screening process involved two independent researchers. Second, we followed ‘PRISMA-ScR’.^49^ This comprised a 20-point checklist, including rationale for the review, justification of methods, explanation of results and a discussion which summarises main results and presents limitations of the study. Despite the rigour in the scoping process, this review also involved some limitations. First, we excluded publications before 2008. Given the dearth of literature in this area, the inclusion of papers pre-2008 may have yielded more results. However, given that practice and policy have evolved over the past decade, it was thought that the inclusion of studies before 2008 may not align with current practice. Second, the review was limited to English language articles, to ensure that all the authors could understand and make informed decisions on the screening process. However, this limited the generalisability of the findings. Moreover, the inclusion of other languages may have added insights about international policies relating to the healthcare assistant in out-of-hours models of care.

## Conclusion

Globally, there is an increasing reliance on the role of the healthcare assistant to deliver care across clinical settings. However, research into their role and contribution remains limited, particularly within the out-of-hours period. The available research suggests that healthcare assistants play an integral role in the delivery of end-of-life and palliative care in the community to patients and families, yet their true contribution and role remains under-researched. Given the priority placed by palliative care patients on the availability of out-of-hours services, the need for further research is this area is warranted. Findings from this scoping review highlight the need for palliative care out-of-hours service providers to consider the role of the healthcare assistant within existing and future models of care.

## Supplemental Material

Supp_File_PRISMA-ScR – Supplemental material for The roles, responsibilities and practices of healthcare assistants in out-of-hours community palliative care: A systematic scoping review Click here for additional data file. Supplemental material, Supp_File_PRISMA-ScR for The roles, responsibilities and practices of healthcare assistants in out-of-hours community palliative care: A systematic scoping review by Anne Fee, Deborah Muldrew, Paul Slater, Sheila Payne, Sonja McIlfatrick, Tracey McConnell, Dori-Anne Finlay and Felicity Hasson in Palliative Medicine
